# Optimizing and developing a scalable, chemically defined, animal component-free lentiviral vector production process in a fixed-bed bioreactor

**DOI:** 10.1016/j.omtm.2023.06.011

**Published:** 2023-07-03

**Authors:** Carme Ripoll Fiol, Marie-Laure Collignon, John Welsh, Qasim A. Rafiq

**Affiliations:** 1 Department of Biochemical Engineering, University College London, Gower Street, WC1E 6BT London, UK; 2 Department of Scientific and Laboratory Services (SLS), Pall Corporation, Reugelstraat 2, 3320 Hoegaarden, Belgium; 3 Department of Research and Development (R&D), Pall Corporation, 5 Harbourgate Business Park, Southampton Road, PO6 4BQ Portsmouth, UK

**Keywords:** animal component-free, chemically defined, serum-free, virus, lentiviral vectors, iCELLis Nano bioreactor, fixed-bed bioreactor, scalability, gene therapy, immunotherapy

## Abstract

Lentiviral vectors (LVVs) play a critical role in gene delivery for *ex vivo* gene-modified cell therapies. However, the lack of scalable LVV production methods and the high cost associated with them may limit their use. In this work, we demonstrate the optimization and development of a scalable, chemically defined, animal component-free LVV production process using adherent human embryonic kidney 293T cells in a fixed-bed bioreactor. The initial studies focused on the optimization of the culture process in 2D static cultures. Process changes such as decreasing cell seeding density on day 0 from 2.5 × 10^4^ to 5 × 10^3^ cells/cm^2^, delaying the transient transfection from 24 to 120 h post-seeding, reducing plasmid DNA to 167 ng/cm^2^, and adding 5 mM sodium butyrate 6 h post-transfection improved functional LVV titers by 26.9-fold. The optimized animal component-free production process was then transferred to the iCELLis Nano bioreactor, a fixed-bed bioreactor, where titers of 1.2 × 10^6^ TU/cm^2^ were achieved when it was operated in perfusion. In this work, comparable functional LVV titers were obtained with FreeStyle 293 Expression medium and the conventional Dulbecco’s modified Eagle’s medium supplemented with 10% fetal bovine serum both at small and large scale.

## Graphical abstract

**Figure:**
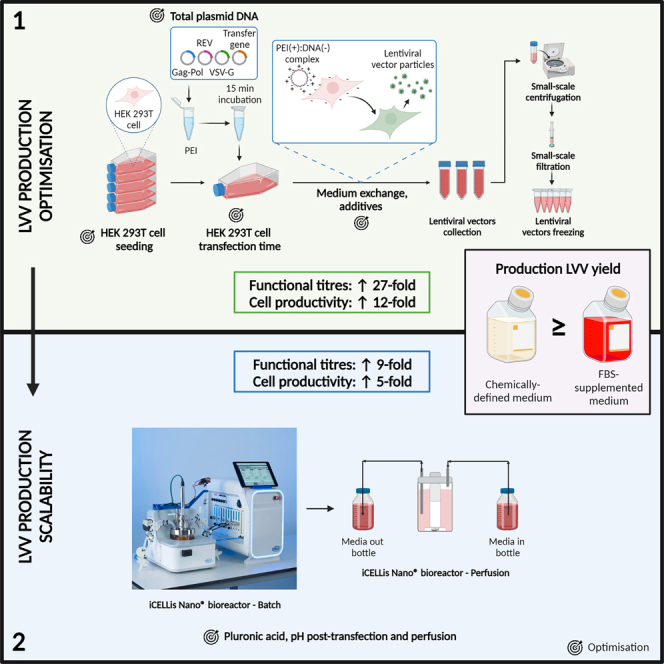

## Teaser

Rafiq and colleagues have demonstrated the optimization and development of a scalable, chemically defined animal component-free manufacturing platform for the production of functional lentiviral vectors (LVVs) using adherent HEK293T cells in a fixed-bed bioreactor. LVVs play a critical role for both gene therapy and viral vaccine applications.

## Introduction

Lentiviral vectors (LVVs) play a critical role in gene delivery for *ex vivo* gene-modified cell therapies. Of the six currently FDA-approved chimeric antigen receptor (CAR)-T cell products, four use LVVs as a vehicle to transfer the CAR gene into the T cells: Kymriah,^1^ Breyanzi,^2^ Abecma,^3^ and Carvykti.^4^ Furthermore, over 300 clinical trials involving the use of LVVs were reported in early 2021^5^ including sickle cell anemia, β-thalassemia, and X-SCID/ADA-SCID.^6^ LVVs are used extensively as a gene delivery vehicle as they have a lower risk of insertional oncogenesis compared with retroviral vectors,^7^ they have the ability to transduce proliferating and non-proliferating cells, and they can integrate into the genome upon cell entry.^8^ In addition, LVVs allow the delivery of relatively high payloads up to 10 kb.^9^

The standard LVV production process relies on the transient transfection of adherent human embryonic kidney 293 (HEK293) cells and their derivatives. Due to the adherent nature of this cell line, the process is usually serum dependent and is carried out in 2D culture systems such as regular T flasks, Corning HYPERflasks or Corning CellSTACK. However, a large-scale good manufacturing practice (GMP)-compliant LVV production process beyond conventional small-scale systems is needed to satisfy the increasing demand from the cell and gene therapy sector.

Single-use fixed-bed bioreactors, such as the iCELLis bioreactor from Pall Corporation or the Scale-X platform from Univercells, provide a promising automated, scalable platform for the generation of large volumes of GMP-grade LVVs using adherent HEK cells. A performance comparison between the iCELLis bioreactor and Scale-X platforms has been published,^10^ where LVVs and adenoviral vector production have achieved similar viral yields when the same culture parameters were used. Other fixed-bed and packed-bed bioreactors such as the BelloCell-500 from CESCO Bioengineering and the Eppendorf Fibra-Cel systems have also been used for viral vector production.^11^^,^^12^

Although fixed-bed bioreactors have already shown great promise achieving high functional LVV titers,^10^^,^^11^^,^^12^ most of the work currently undertaken in the field relies on serum-containing medium formulations as they are associated with higher LVV titers and increased LVV stability. Removing animal-derived serum from the process would not only reduce long-term process cost and supply chain issues, but also decrease batch-to-batch variability and reduce manufacturing risks.^13^^,^^14^ Adherent HEK293T cells have been adapted to suspension cultures by removing animal-derived serum from the medium and using serum-free medium formulations for LVV production.^15^ However, the adaptation from adherent to suspension cell cultures is cumbersome and frequently results in a delay in the clinical phase. Moreover, the optimization and scale-up of suspension cell cultures in stirred-tank bioreactors (STRs) tend to be more intricate compared with adherent cultures due to the sensitivity of LVVs, as enveloped virus, to the shear stress levels inherent in aerated STRs. In addition, the implementation of perfusion and the downstream processing of these viruses necessitates an additional clarification step to eliminate suspended cells, further contributing to the complexity of these processes.

The iCELLis platform has been used for a wide range of applications such as viral vaccines,^16^ poxvirus production on chicken embryo fibroblasts,^17^ VERO cell line metabolism studies,^18^ and culture of insect cells.^19^ However, the platform is routinely used for virus production such as adenovirus,^20^ retrovirus,^21^^,^^22^ AAV,^23^^,^^24^ and LVVs^25^^,^^26^ using mainly transient transfection of adherent cell lines such as HEK293, HEK293T, or HEK293T/17 cells. A study by Powers et al.^27^ demonstrated the culture of a stable packaging cell line in the iCELLis Nano bioreactor for the production of LVVs. In the study, the fetal bovine serum (FBS) concentration, pH post-induction, and day of induction were explored in an effort to optimize the scale-up production of GPRTG-EF1α-hγ_c_-OPT LVVs. However, there have been no peer-reviewed studies that demonstrate the use of an adherent bioreactor platform such as the iCELLis Nano for animal component-free production of LVVs using HEK293T cells.

The work presented in this study demonstrates, for the first time, the optimization and development of a scalable, chemically defined animal component-free manufacturing platform for the production of highly functional LVV titers using adherent HEK293T cells in a fixed-bed bioreactor. The initial studies focus on optimizing the culture conditions in a 2D static culture system, with a focus on seeding density, transfection day, total plasmid DNA concentration, and supplementation of additives, before the optimized process is transferred to the iCELLis Nano bioreactor. We hypothesized that using an animal component-free medium such as FreeStyle 293 Expression medium, designed for suspension cultures could generate comparable LVV production yields as conventional Dulbecco’s modified Eagle’s medium (DMEM) supplemented with 10% FBS in 2D cultures and in the iCELLis Nano bioreactor.

## Results

The production of third-generation vesicular stomatitis virus glycoprotein (VSVG)-pseudotyped LVVs encoding for a green fluorescent protein (GFP) gene was optimized. In this study, we focused initially on optimizing the culture conditions for a 2D culture process as part of a three-phase optimization process, investigating (1) seeding density and transfection time, (2) total plasmid DNA concentration, and (3) medium exchange pre/post-transfection and the provision of additives to compensate for the lack of FBS, before taking these optimized conditions and transferring the process to the iCELLis Nano bioreactor. The LVV production optimization at small scale in T75 flasks was performed using FreeStyle 293 Expression medium, a chemically defined, animal component-free medium with no animal-derived components, originally designed for suspension cultures. Following optimization, a side-to-side comparison of LVV production was undertaken using FreeStyle 293 Expression medium and the standard DMEM supplemented with 10% FBS, before the process was transferred to the iCELLis Nano bioreactor. A summary of all experimental conditions performed at small scale and the outcomes can be found in Table 1.

**Table 1:** Experimental conditions and outputs for all experiments performed at small scale to optimize LVV production both in animal component-free and serum-containing conditions

	Experimental conditions					Outputs					
	Seeding density (cells/cm^2^)	Transfection time post-seeding (h)	Total plasmid DNA (ng/cm^2^)	Medium exchange	5 mM sodium butyrate	Functional titers (TU/cm^2^)	Increase from previous protocol	Increase from unoptimized	Cell productivity (TU/cell)	Increase from previous protocol	Increase from unoptimized
Unoptimized	2.5 × 10^4^	48	333	before transfection	no	1.0 × 10^6^	1.0×	1.0×	7.9	1.0×	1.0×
Phase I	5 × 10^3^	120	333	before transfection	no	4.6 × 10^6^	4.6×	4.6×	17.4	2.2×	2.2×
Phase II	5 × 10^3^	120	167	before transfection	no	7.5 × 10^6^	1.6×	7.5×	28.6	1.6×	3.6×
Phase III	5 × 10^3^	120	167	after transfection	yes	2.1 × 10^7^	2.8×	20.6×	79.7	2.8×	10.1×
Final studies – FreeStyle 293 Expression medium	5 × 10^3^	120	167	after transfection	yes	2.7 × 10^7^	–	26.9×	93.3	–	11.8×
Final studies – DMEM + 10% FBS	5 × 10^3^	120	167	after transfection	yes	3.7 × 10^7^	–	–	85.5	–	–

DMEM, Dulbecco’s modified Eagle medium; FBS, fetal bovine serum; TU, transducing units. “–” indicates comparison is not appropriate.

### Phase I: Investigating the impact of seeding density and transfection time on cell growth kinetics and LVV production yields

**Figure 1:**
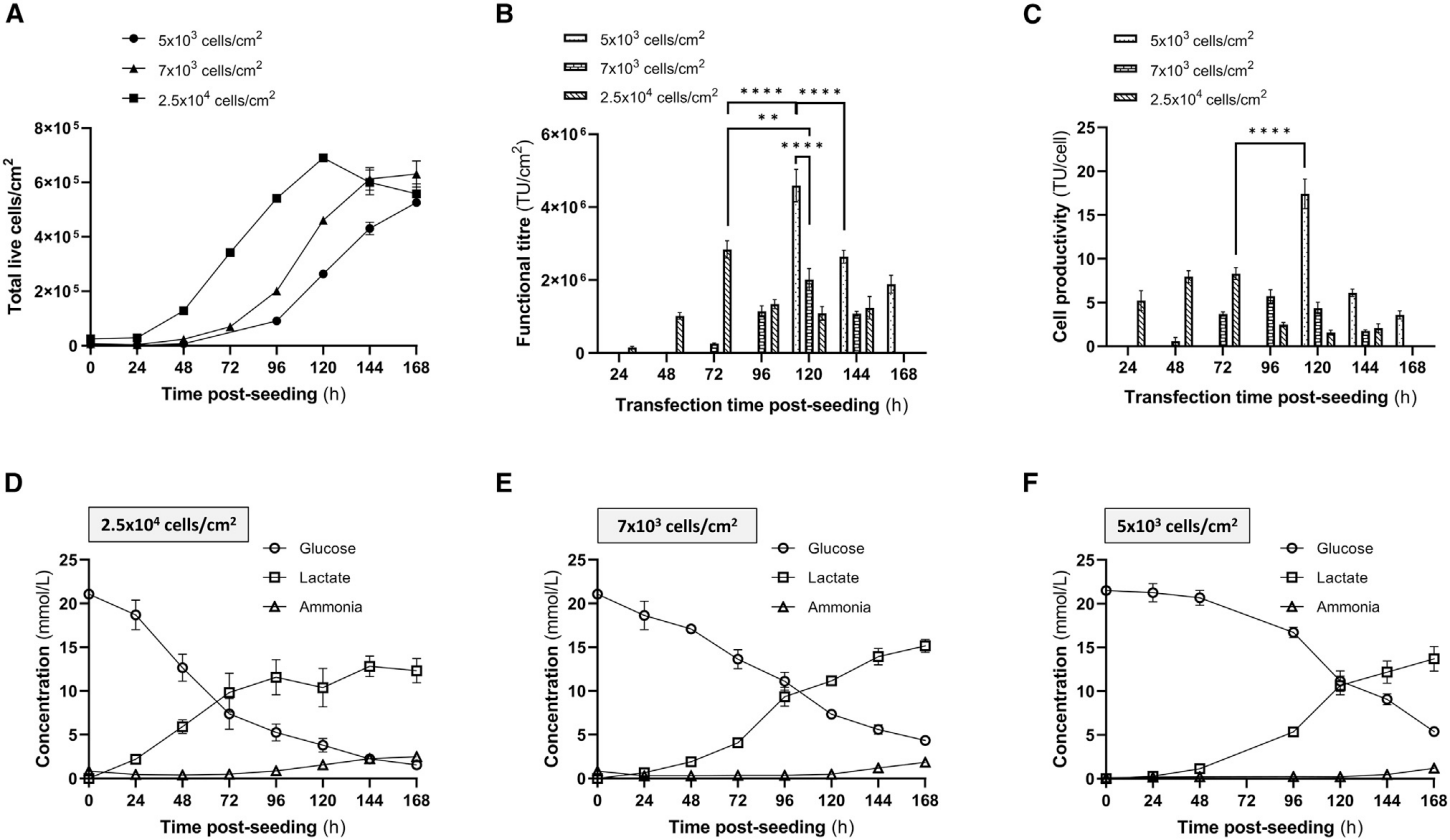
Cell growth kinetics, LVV production yields, and metabolite analysis phase I of the optimization (A) Cell growth (live cells/cm^2^) after seeding 5 × 10^3^, 7 × 10^3^, and 2.5 × 10^4^ cells/cm^2^, (B) functional LVV titers (TU/cm^2^), and (C) cell productivity (TU/cell) after seeding the aforementioned three seeding densities and transfecting cells with a total plasmid DNA concentration of 333 ng/cm^2^. (D–F) Glucose consumption and lactate/ammonia production for the different seeding densities 2.5 × 10^4^ cells/cm^2^ (D), 7 × 10^3^ cells/cm^2^ (E), and 5 × 10^3^ cells/cm^2^ (F) during the experiments and measured daily from the cell culture medium. Data of results are shown as mean values ±SD of n = 3 technical replicates. Statistical analysis was performed using ordinary one-way ANOVA with Tukey’s multiple comparisons test and significance is shown when p values were ∗∗p ≤ 0.01, ∗∗∗∗p ≤ 0.0001. Only relevant statistical analysis is shown.

In phase I, three different HEK293T cell seeding densities were explored in FreeStyle 293 Expression medium: 5 × 10^3^, 7 × 10^3^, and 2.5 × 10^4^ cells/cm^2^. For each of the seeding densities, different transfection times were assessed but a constant harvest time of 48 h post-transfection was chosen for all experiments. Usually, cell transfection should occur during the exponential phase of the growth curve,^28^ which in this case was between 24 and 72 h when 2.5 × 10^4^ cells/cm^2^ were seeded and between 24 and 120 h when 5–7 × 10^3^ cells/cm^2^ were seeded as illustrated in Figure 1A. The highest functional LVV titers were found when transfection was done 72 h after seeding 2.5 × 10^4^ cells/cm^2^ and 120 h after seeding either 7 × 10^3^ or 5 × 10^3^ cells/cm^2^ (Figure 1B). The maximum functional LVV titer obtained across all conditions was the condition where 5 × 10^3^ cells/cm^2^ were seeded followed by transfection at 120 h. This also correlated with the highest HEK293T cell productivity (Figure 1C). In this condition, 4.6 × 10^6^ transducing units (TU)/cm^2^ were achieved with a cell productivity of 17.4 TU/cell.

Metabolite measurements were obtained via daily sampling during the culture period. No glucose depletion or lactate and ammonia inhibition were observed in any of the seeding densities until the transfection time points (Figures 1D–1F).

### Phase II: Investigating the impact of total plasmid DNA concentration on functional LVV titers and cell productivity

**Figure 2:**
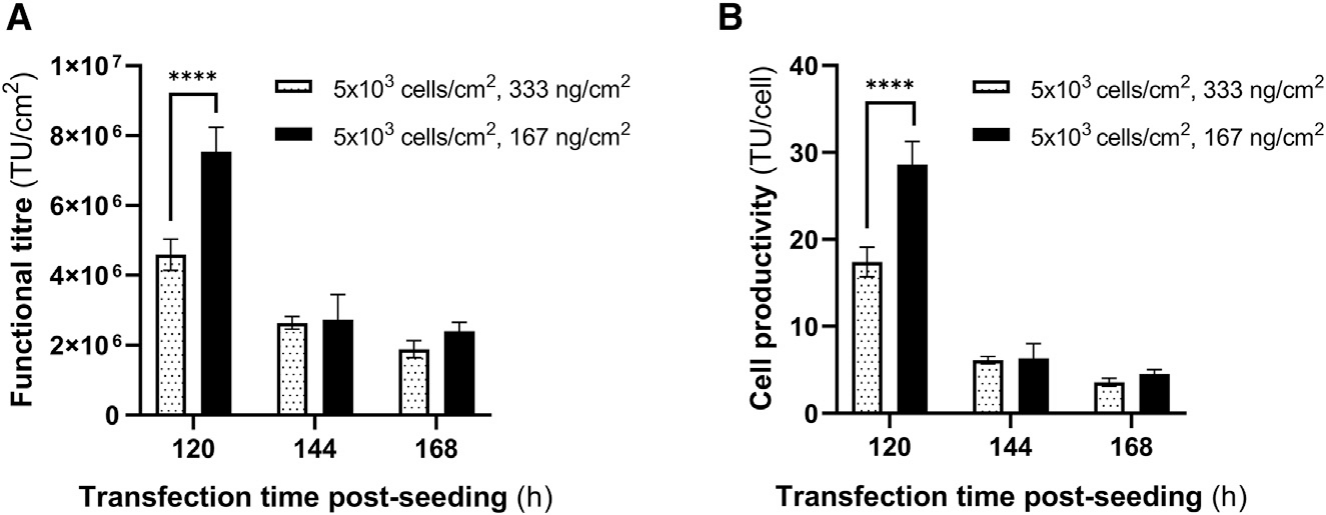
LVV production yields during phase II of the optimization (A) Functional LVV titers (TU/cm^2^) and (B) cell productivity (TU/cell) after seeding 5 × 10^3^ cells/cm^2^ using two different total plasmid DNA concentration concentrations. Data of results are shown as mean values ± SD of n = 3 technical replicates. Statistical analysis was performed using ordinary two-way ANOVA with Tukey’s multiple comparisons test and significance is shown when p values were ∗∗∗∗p ≤ 0.0001. Only relevant statistical analysis is shown.

In the original unoptimized process, a total plasmid DNA concentration of 333 ng/cm^2^ was used. In phase II of the optimization, a 2-fold reduction of the total plasmid DNA concentration, 167 ng/cm^2^, was explored. Building on the results from the previous studies, an inoculation density of 5 × 10^3^ cells/cm^2^ was selected. Comparing the two total plasmid DNA concentration values (333 vs. 167 ng/cm^2^), the highest LVV titers were found at 120 h matching the findings from phase I (Figure 2A). Using a total plasmid DNA concentration of 167 ng/cm^2^, half of the concentration used previously, 7.5 × 10^6^ TU/cm^2^ of LVVs were obtained (Figure 2A). These functional titers were 1.6-fold higher than those generated using double the amount of DNA (Figure 2A). Similarly, a 1.6-fold higher cell productivity was also observed when the lower DNA concentration was used (Figure 2B).

### Phase III: Investigating the impact of supplementing additives, provision of sodium butyrate, and medium exchange timings

The combination of non-essential amino acids, sodium pyruvate, and chemically defined lipids, from here onward referred to as “additives,” and, separately, sodium butyrate was explored in phase III of the optimization to compensate for the lack of FBS in the medium during the LVV production. This combination of additives was explored because Leinonen et al.^26^ demonstrated increased productivity of the process upon a medium exchange post-transfection using this combination of additives. In our study, additives were added either from the beginning of the culture on day 0 (pre-transfection) or 6 h after transfection (post-transfection). The point at which a medium exchange was performed was also investigated. In the original process, the medium exchange was performed before the addition of the DNA/PEI complexes in the flasks. However, we decided to undertake an investigation comparing the effect of performing a medium exchange both pre- and post-transfection. This was undertaken from a bioprocessing standpoint as it was hypothesized that a medium exchange 6 h post-transfection would minimize any residual DNA and PEI being passed onto the downstream processing steps.

**Figure 3:**
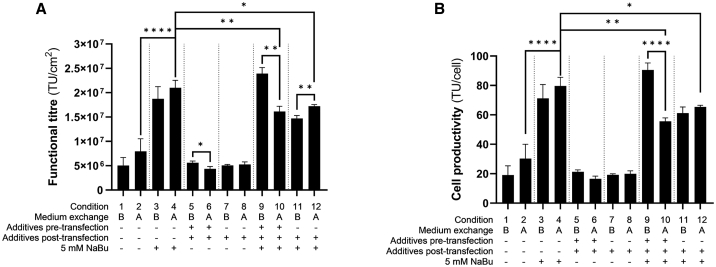
LVV production yields during phase III of the optimization (A) Functional LVV titers (TU/cm^2^) and (B) cell productivity (TU/cell) after seeding 5 × 10^3^ cells/cm^2^ when medium was exchanged before addition of DNA/PEI complexes (B) or 6 h after the transfection (A). Additives were added either from the beginning of the culture on day 0 (pre-transfection) or 6 h after transfection (post-transfection). Additives, non-essential amino acids + chemically defined lipids + sodium pyruvate; NaBu, sodium butyrate. Data of results are shown as mean values ± SD of n = 3 technical replicates. Statistical analysis was performed using ordinary one-way ANOVA with Tukey’s multiple comparisons test and significance is shown when p values were ∗p ≤ 0.05, ∗∗p ≤ 0.01, ∗∗∗∗p ≤ 0.0001; ns, not significant. Only relevant statistical analysis is shown.

Figures 3A and 3B demonstrate that performing a medium exchange post-transfection as opposed to pre-transfection had either no effect on LVV titers or increase them in some cases (conditions 1 vs. 2, 3 vs. 4, 7 vs. 8, and 11 vs. 12, Figure 3A). In two cases, condition 5 vs. 6 and 9 vs. 10, LVV titers decreased upon medium exchange post-transfection. This is believed to be caused by the presence of additives during the transfection step. Additives supplemented pre-transfection were not washed out as the medium was not exchanged prior to transfection, thereby potentially interfering in the transfection process.

The addition of 5 mM sodium butyrate 6 h post-transfection led to the production of 2.1 × 10^7^ TU/cm^2^ when medium was exchanged after transfection (condition 4, Figure 3A). This is more than 2-fold increase compared with the same condition without sodium butyrate (condition 2). The supplementation of additives (a combination of non-essential amino acids, chemically defined lipids, and sodium pyruvate) did not have a positive impact regardless of the moment of the addition during the production in the conditions tested using FreeStyle 293 Expression medium (conditions 6 and 8). It was believed that the impact on increasing the titers when the additives were used in combination with sodium butyrate (conditions 11 and 12) was from the latter and therefore this was the key compound to be added in chemically defined, animal component-free medium LVV production. Similar trends were observed in the HEK293T cell productivity in these experiments (Figure 3B).

### Comparable LVV production yields can be achieved using FreeStyle 293 Expression medium and a serum-containing medium in small-scale 2D static culture

To evaluate the optimized protocol in T75 static small scale, a side-to-side LVV production process using DMEM supplemented with 10% FBS and FreeStyle 293 Expression medium was performed. From the previous phases of the optimization process, the optimal conditions were found to be seeding 5 × 10^3^ HEK293T cells/cm^2^ on day 0, transfecting 120 h later with 167 ng/cm^2^ of total plasmid DNA concentration, and performing a complete medium exchange 6 h post-transfection with 5 mM sodium butyrate addition.

**Figure 4:**
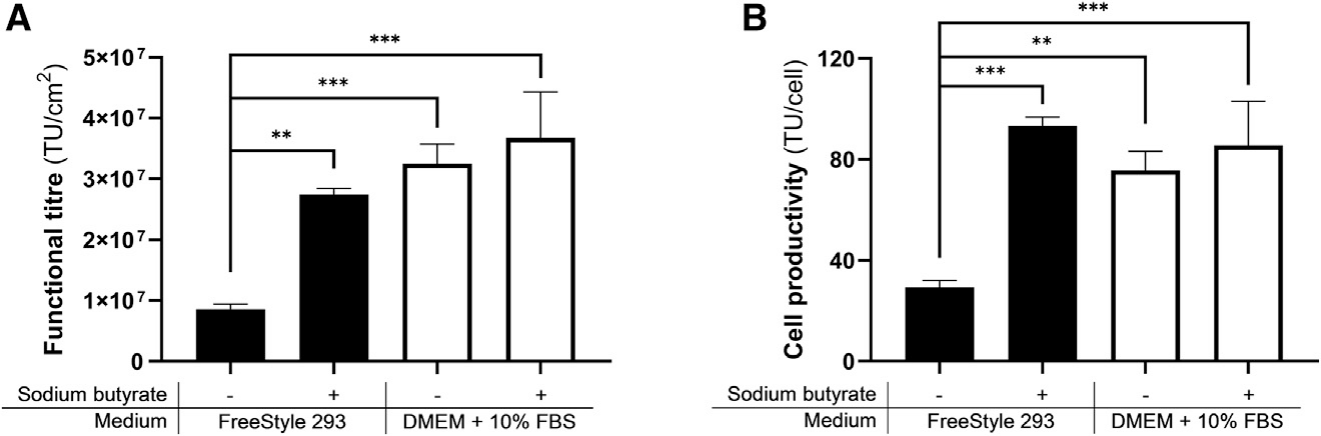
Functional titers during the final studies of the optimization (A) Functional LVV titers (TU/cm^2^) and (B) cell productivity (TU/cell) after seeding 5 × 10^3^ cells/cm^2^. Data of results are shown as mean values ± SD. Statistical analysis was performed using ordinary two-way ANOVA with Tukey’s multiple comparisons test and significance is shown when p values were ∗∗p ≤ 0.01, ∗∗∗p ≤ 0.001.

Under optimal conditions, 2.7 × 10^7^ TU/cm^2^ were achieved when FreeStyle 293 Expression medium was used, resulting in more than 3-fold increase compared with the same protocol without sodium butyrate addition (Figure 4A). The addition of the sodium butyrate was key when the animal component-free medium was used; however, during the LVV production with DMEM supplemented with 10% FBS, the sodium butyrate addition did not result in a statistical difference in functional LVV titers. There was no statistical difference between the functional LVV titers achieved with the optimized conditions in the animal component-free medium and any of the conditions with DMEM supplemented with 10% FBS. Similarly, the cell productivity of the HEK293T cells was comparable with respect to LVV production with the FreeStyle 293 Expression medium and the DMEM supplemented with 10% FBS (Figure 4B).

### Cell growth and metabolite analysis in the iCELLis Nano bioreactor

The chemically defined, animal component-free LVV production process optimized in 2D static culture, which generated LVV titers comparable to serum-containing medium, was then transferred to the iCELLis Nano bioreactor.

Eight runs were successfully completed in the iCELLis Nano bioreactor with an appropriate T-flask control for each condition. HEK293T cells were cultured in FreeStyle 293 Expression medium (runs 1 to 7) or in DMEM supplemented with 10% FBS (run 8) for 6 or 7 days in a 0.53 m^2^ vessel. This vessel size corresponds to a low-compaction, 2 cm fixed-bed height.

In run 1, 2.5 × 10^4^ cells/cm^2^ were inoculated following pre-optimization conditions in flasks, whereas in the rest of the runs 5 × 10^3^ cells/cm^2^ were inoculated. For all runs, cells were inoculated on day 0 with a final working volume of 850 mL, transfected on day 2 (run 1) or day 5 (runs 2 to 8), and lentiviral particles were harvested 48 h post-transfection on day 4 or day 7 depending on the run. The parameters and culture conditions in the different bioreactor runs are summarized in Table 2.

**Table 2:** iCELLis Nano bioreactor parameters and culture conditions for the different runs

Run	Medium	Seeding density (cells/cm^2^)	Transfection time post-inoculation (days)	Total plasmid DNA (ng/cm^2^)	DNA/PEI complexation medium	pH post-T	Medium exchange	0.1% Pluronic F-68 acid	5 mM NaBu	Mode
1	FreeStyle 293	2.5 × 10^4^	2	333	FreeStyle 293	7.2	before transfection	no	no	batch
2	FreeStyle 293	**5 × 10**^**3**^	**5**	**167**	FreeStyle 293	7.2	**after transfection**	no	**yes**	batch
3	FreeStyle 293	5 × 10^3^	5	167	FreeStyle 293	7.2	after transfection	**post-T**	Yes	batch
4	FreeStyle 293	5 × 10^3^	5	167	FreeStyle 293	7.2	after transfection	**pre- and post-T**	yes	batch
5	FreeStyle 293	5 × 10^3^	5	167	FreeStyle 293	6.8	after transfection	post-T	yes	batch
6	FreeStyle 293	5 × 10^3^	5	167	**DMEM**	**6.8**	after transfection	post-T	yes	batch
7	FreeStyle 293	5 × 10^3^	5	167	FreeStyle 293	6.8	after transfection	post-T	yes	**perfusion**
8	**DMEM + 10% FBS**	5 × 10^3^	5	167	FreeStyle 293	6.8	after transfection	post-T	yes	perfusion

In bold, changes performed in that particular run compared with the previous one. DMEM, Dulbecco’s modified Eagle’s medium; NaBu, sodium butyrate; pre-T, pre-transfection; post-T, post-transfection.

**Figure 5:**
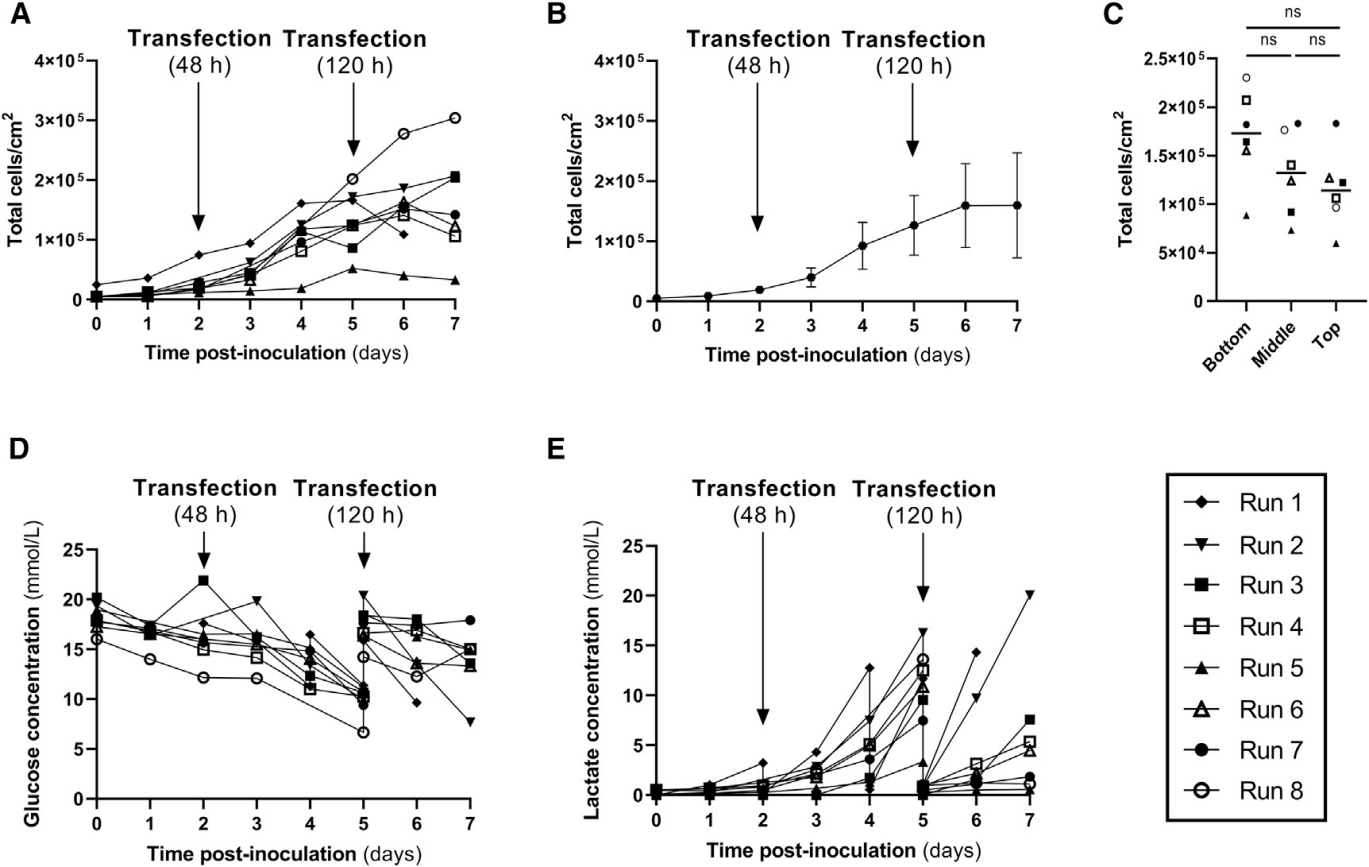
Cell growth kinetics and metabolite analysis in the iCELLis Nano bioreactor (A) Cell growth (total live cells/cm^2^) after seeding 2.5 × 10^4^ cells/cm^2^ in run 1 or 5 × 10^3^ cells/cm^2^ for runs 2 to 8, (B) average cell growth in runs 2 to 8, (C) cells distribution in the fixed-bed of the iCELLis Nano bioreactor by the end of runs 3 to 8, (D) glucose levels, and (E) lactate production for the different bioreactor runs. Transfection at 48 h corresponds to run 1, while transfection at 120 h corresponds to runs 2 to 8. Data represented correspond to eight different iCELLis Nano runs. For each time point in (A and C), three different macrocarriers were sampled. Statistical analysis was performed using ordinary one-way ANOVA with Tukey’s multiple comparisons test (ns, not significant).

Daily sampling of three macrocarriers from the top of the fixed-bed allowed the assessment of cell growth in the bioreactor. In all runs, cell growth was observed over the culture period (Figure 5A). However, there were differences in cell growth despite the same seeding density with higher variability observed in the later time points of the experiments (Figure 5B). Cell growth in the control flasks was consistent and similar cell numbers were achieved by the end of each process (data not shown)*.* In run 8, performed using DMEM supplemented with 10% FBS instead of FreeStyle 293 Expression medium, the highest cell density was achieved.

For runs 2 to 8, there was an average of 1.3 × 10^5^ cells/cm^2^ at the moment of transfection on day 5 and, in general, cells continued growing after transfection until the end of the run. In run 1, cell density was 7.5 × 10^4^ cells/cm^2^ at the moment of transfection, lower than in the rest of the runs due to earlier transfection on day 2 instead of on day 5. In run 5 cells did not grow as much as in previous runs and, therefore, there were less cells at the moment of transfection and cell numbers started to decrease shortly after transfection.

Macrocarriers from the top, middle, and bottom of the fixed-bed were sampled at the end of the run to assess the distribution of the cells across the bed. A higher average number of cells in the bottom carriers was consistently found by the end of the runs, while similar average cell numbers were present in the middle and top carriers (Figure 5C). On average, there were approximately 45% more cells in the bottom carriers than in the top carriers, similar to that reported previously.^25^

Glucose was consumed during the expansion phase in all runs until the moment of transfection, when a medium exchange was performed either before transfection (run 1) or 6 h later (runs 2 to 8) (Figure 5D). The lowest level of glucose was found in run 8 (6.65 mmol/L), the run with higher cell growth and also the only run where DMEM was used a basal medium, which has a lower starting glucose level than FreeStyle 293 Expression medium. Notably, cells never consumed all of the available glucose during the experiments, nor during the 5 days of cell expansion without medium feed. This is likely to be because a low cell seeding density was used during inoculation for these runs and cell densities achieved were not at a level to result in significant glucose limitations. In run 1, LVV harvests were done after 48, 72, and 96 h post-transfection and therefore glucose levels were restored after the whole medium exchange for harvesting.

The lactate concentration in each run increased, as expected, until the point of transfection, but never reached higher values than 15–20 mM (Figure 5E). LVV harvests were undertaken 48 h post-transfection for all runs, generally on day 7. In the perfusion runs (runs 7 and 8) fresh medium was perfused and thus the level of glucose remained high and lactate levels were kept to minimum. As mentioned previously, cells did not grow in run 5 and, accordingly, lactate levels in this run were consistently lower than all other runs in batch mode.

### An animal component-free LVV production process comparable with conventional serum-containing LVV production was achieved in the iCELLis Nano bioreactor

Two iCELLis Nano bioreactor runs in batch mode were performed initially with FreeStyle 293 Expression medium: one with the unoptimized conditions as a baseline run (run 1) and one using the optimized conditions from the 2D static culture studies (run 2). Further optimization in batch was performed in the bioreactor in an attempt to further increase functional LVV titers, total physical LVV particles (VP), and HEK293T cell productivity. Pluronic F-68 acid (runs 3 and 4), pH post-transfection (run 5), and PEI:DNA complexation medium (run 6) were explored in these additional runs. The optimized batch bioreactor process was then operated in perfusion (run 7), which is considered the final animal component-free run and also used for comparison with the standard LVV production using DMEM supplemented with 10% FBS.

**Figure 6:**
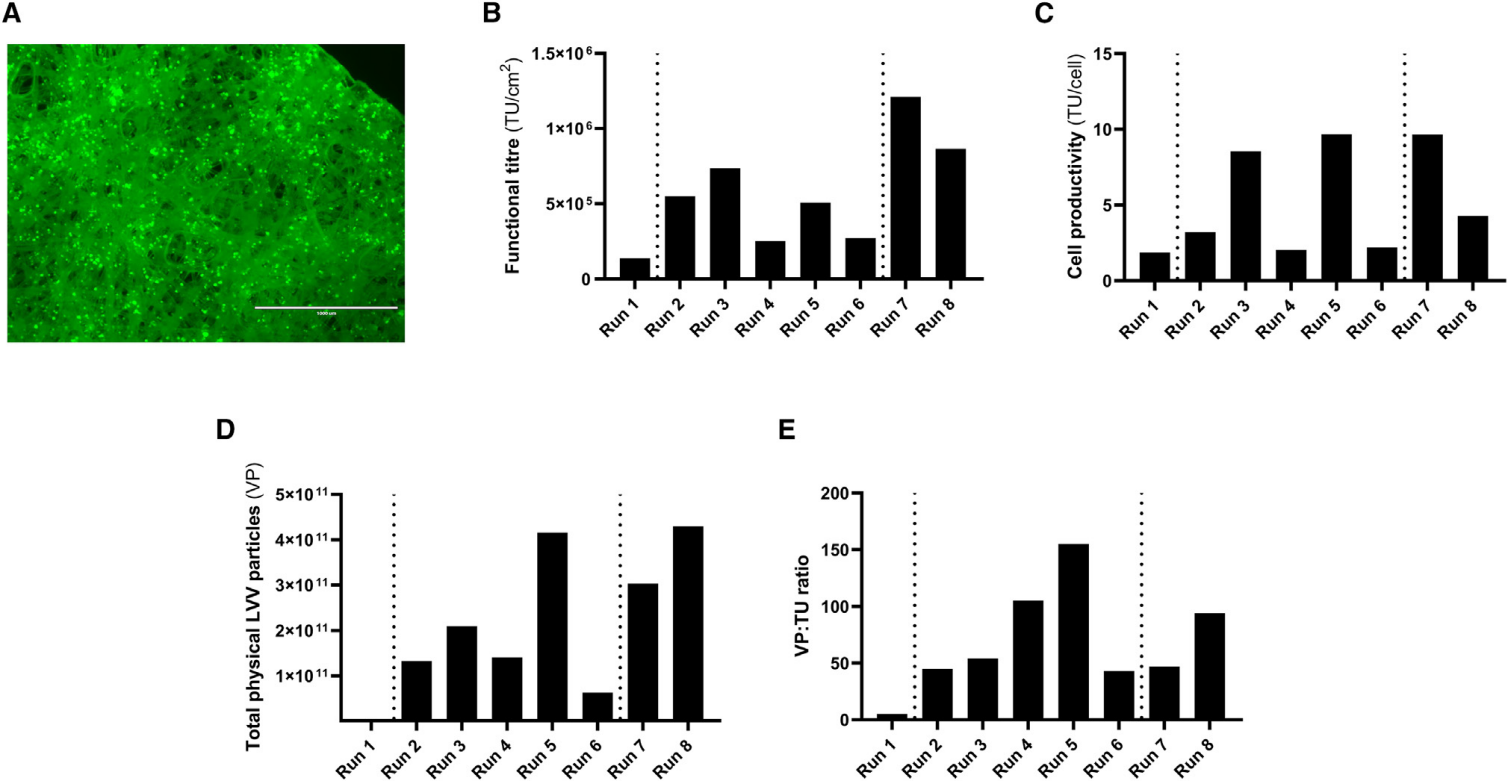
LVV production yields in the iCELLis Nano bioreactor (A) Fluorescent microscopy image of a macrocarrier sampled from the fixed-bed after GFP transfection, (B) functional LVV titers (TU/cm^2^), (C) cell productivity (TU/cell), (D) total physical LVV particles, and (E) VP/TU ratio in the different bioreactor runs. Dashed division lines indicate (left to right) runs using pre-optimization conditions, runs in batch mode, and runs in perfusion mode. TU, transducing units; VP, total physical LVV particles. Scale bar, 1,000 mM in (A).

An initial assessment of the transfection was performed by evaluating the upper macrocarriers from the fixed-bed under the fluorescent microscope (Figure 6A). The presence of positive GFP cells in the carriers indicated a successful transfection, despite not being a quantitative measurement.

The functional LVV titers from the viral harvest were assessed by the transduction of HEK293T cells with different serial dilutions of the unconcentrated virus supernatant. The functional titers were normalized to the 5,300 cm^2^ production surface in an attempt to increase comparability across different studies. In run 2, where the optimized conditions at small scale were used, the titers were 4× higher than in run 1, where unoptimized conditions were used (Figure 6B). As the functional LVV titers were lower than in control flasks, Pluronic F-68 acid was added post-transfection in run 3 or both pre- and post-transfection in run 4 in an attempt to increase functional titers by protecting the HEK293T cells and the LVV particles from shear in the bioreactor.^29^^,^^30^ The highest titers were found in run 3, achieving 7.4 × 10^5^ TU/cm^2^, as opposed to the 2.5 × 10^5^ TU/cm^2^ achieved in run 4.

It was described previously^27^^,^^31^ that mildly acidic conditions post-transfection favor the production of LVVs; thus, in run 5 this was explored achieving functional titers of 5.1 × 10^5^ TU/cm^2^ (Figure 6B). An additional run (run 6) was performed combining the pH 6.8 post-transfection and using DMEM instead of FreeStyle 293 Expression medium for the DNA/PEI complexation step. The use of DMEM for the complexation decreased the functional LVV titers to 2.7 × 10^5^ TU/cm^2^.

In run 7, the operation of the bioreactor was then switched from batch to perfusion at one vessel volume per day (1 vvd) in an attempt to further increase the functional titers by constantly supplying fresh nutrients, depleting cellular waste from the medium and harvesting the LVVs at 4°C. Accordingly, the LVV harvest bottle was kept in a bucket with ice to maintain the viral suspension at 4°C. Perfusion led to the highest functional LVV titers of all runs, achieving 1.2 × 10^6^ TU/cm^2^; hence, conditions in this run were considered optimal when FreeStyle 293 Expression medium was used during the LVV production (Figure 6B).

As the primary aim of this work was to generate comparable LVV production yields using a chemically defined, animal component-free process instead of the standard DMEM supplemented with 10% FBS in the bioreactor, a final run was performed with the same conditions as run 7 but using DMEM supplemented with 10% FBS. In this run, run 8, functional LVV titers of 8.7 × 10^5^ TU/cm^2^ were achieved, demonstrating that similar titers if not higher can be obtained with both animal component-free and serum-containing media (Figure 6B).

The cell productivities for each run were calculated using the total TU and the total number of cells at the moment of transfection. The highest cell productivities were found in runs 3, 5, and 7, where between 8.6 and 9.7 TU/cell were produced (Figure 6C). Remarkably, cell productivity was higher in run 7 when FreeStyle 293 Expression medium was used as opposed to cell productivity in run 8 where the conventional DMEM supplemented with 10% FBS was used.

In general, the total VP trend (Figure 6D) did not always correlate with the functional LVV titers in each bioreactor run (Figure 6B). While the highest functional titers were found in run 7, the highest number of total VP were generated in runs 5 and 8. However, in run 5 the ratio between VP and TU was higher than in the rest of the runs (Figure 6E).

## Discussion

The use of LVVs has been increasing over the last decade and it has been demonstrated that they play a key role in gene delivery with the number of LVV clinical trials increasing over time. It is expected that the amount of LVVs required will further increase with the commercialization of some gene therapies currently in clinical trials. Yet, the lack of scalable LVV production methods and the high cost associated with them may limit their use. In this work, we have developed a scalable, chemically defined, animal component-free LVV production process using adherent HEK293T cells in a fixed-bed bioreactor achieving functional LVV titers of 1.2 × 10^6^ TU/cm^2^, comparable if not higher to those achieved with conventional DMEM supplemented with 10% FBS.

Initially, the production was optimized at small-scale exploring HEK293T cell seeding density, day of transfection, total plasmid DNA concentration, and additives supplementation in the animal component-free process. A low HEK293T cell seeding density is of benefit for larger-scale processes, reducing the seed train complexity due to fewer cells needed during the inoculation. In this study, it was demonstrated that a seeding density of 5 × 10^3^ cells/cm^2^ led to the highest functional LVV titers at small scale when compared with 7 × 10^3^ or 2.5 × 10^4^ cells/cm^2^. This low seeding density was recently used in the iCELLis Nano bioreactor to generate oncolytic H-1 protoparvovirus for cancer treatments,^32^ but was never previously used for LVV production in which the lowest seeding density published was 7 × 10^3^ cells/cm^2^.^25^ The reduction in the inoculation cell density resulted in a delay in transfection from 48 to 120 h, but we considered that an overall process of 7 days is an acceptable process length for the production of these viral vectors. According to the data obtained, it would take a total of 92 days with the shorter unoptimized 3 day process to obtain the same functional LVV titers than those obtained with the optimized 7 days process.

The functional LVV titers in the small-scale experiments were further increased by reducing the total plasmid DNA concentration to 167 ng/cm^2^. As the DNA and the PEI amounts were added as a ratio of DNA/PEI 1:2.75 (w/w), the increase in functional LVV titers was likely caused by the reduction of PEI being added to the culture, known to have a cytotoxic effect on the cells.^33^ Although PEI:DNA molecules have been reported less toxic than free PEI molecules,^34^ there is still a risk of free PEI molecules after the complexation increasing cytotoxicity. As the total plasmid DNA concentration is one of the main driving costs for LVV production,^35^ decreasing the total plasmid DNA concentration not only had a positive impact reducing cell cytotoxicity but also improved the cost-effectiveness of the process developed. Another potential cause for improved LVV production yields when a lower total plasmid DNA concentration was used could be a more suitable DNA and PEI concentration during the complexation. The concentration of DNA and PEI in the complexation solution can affect the size of the transfection particles; therefore, impacting on the transfection efficiency and yields. In our study, the total complexation volume remained unaltered when the total plasmid DNA concentration was decreased, so the DNA and PEI concentration decreased.

Moreover, it is important that the residual DNA and PEI post-transfection are minimized to avoid adding complexity to the DSP. In this work, changing the medium after transfection did not have an impact on LVV titers compared with changing it before transfection in most of the conditions assessed and was therefore preferred. When additives (non-essential amino acids, chemically defined lipids, and sodium pyruvate) were supplemented at the beginning of the culture and were present during transfection, the medium exchange after transfection resulted in lower titers compared with changing it before transfection. It is believed that this is caused by the presence of these additives during transfection, as it is been described previously that the composition of the medium has a huge impact on transfection rates and small changes can cause large impacts on viral titers.^36^^,^^37^

The supplementation of sodium butyrate demonstrated a significant impact on the chemically defined, animal component-free LVV production by increasing yields by 3-fold. Our results are consistent with previously published studies,^38^^,^^39^^,^^40^^,^^41^ although it was also earlier reported that this increment can vary depending on the different DNA constructs.^42^ The combination of additives (sodium pyruvate + chemically defined lipids + non-essential amino acids) did not have an impact in the LVV production yields, contrary to what was published by Leinonen et al*.*^26^ In another study, the supplementation of a serum-containing medium with cholesterol has shown increases in the VSVG-pseudotyped LVV titers, hypothesized to be due to the alteration on the lipid composition of the viral vectors due to changes in the composition of the producer cell membrane where LVVs bud.^43^ In the aforementioned studies, DMEM was used as a basal medium instead of FreeStyle 293 Expression medium and it is likely that the impact of the additives supplementation vary depending on the basal medium used.

Although we are aware that other parameters could have been optimized at small scale, such as ratio of different plasmid DNA, PEI/DNA complexation medium, time and temperature, or viral supernatant collection time, the production yields were considered relevant for cell and gene therapy applications and the process was considered optimized at small scale. The process was then transferred to the iCELLis Nano bioreactor. This 1 L fixed-bed bioreactor is an effective process development platform as the process can be scaled to the larger version of the platform, the iCELLis 500 bioreactor.^26^

In this work, we have demonstrated that it is feasible to culture adherent HEK293T cells in FreeStyle 293 Expression medium in the iCELLis Nano bioreactor. The differences in cell growth in the latter time points of the experiments, especially on days 6 and 7, were potentially caused by the differences in culture and experiment conditions. For instance, the decrease in pH post-transfection in runs 5 to 8 may have increased functional titers but at the expense of an impact on cell growth. The cell growth in run 5 was notably lower compared with other runs, exhibiting a consistently low cell count throughout the entire culture duration. However, a plausible explanation for the low cell growth in that run has not yet been identified, highlighting the need for further exploration of potential factors affecting cell viability and proliferation in the bioreactor. Previously, differences in cell growth in the bioreactor and in the different sections of the fixed-bed were reported by Valkama et al.^25^ Although they have reported that differences in the distribution of the cells in the fixed-bed were higher in high-compaction fixed-bed bioreactors compared with low-compaction fixed-beds, in our study the differences were still present in the low-compaction 0.53 m^2^ fixed-bed. Similar findings were shown in Leinonen et al.,^10^ where 2- to 3-fold more cells were found in the middle carriers compared with the bottom ones.

In our work, the total plasmid DNA concentration used for each run was calculated based on the production surface. Hence, the different cell density during the transfection for each run also impacted the amount of DNA per 10^6^ cells. At transfection time some runs had fewer cell numbers, potentially resulting in a higher PEI concentration per cell and therefore reducing functional LVV titers due to cell cytotoxicity by the PEI. To avoid differences in functional LVV titers due to different cell growth, cell productivity was calculated as a normalized parameter to compare across runs.

During the bioreactor work, several modifications to the optimized process at small scale were explored in an attempt to increase functional LVV titers. Although Pluronic F-68 acid is widely used to stabilize the cell-liquid interface,^44^ its addition post-transfection in run 3 did not have a large impact in increasing functional titers per surface area compared with previous run 2. Despite that, a trend for enhanced cell productivity compared with previous runs was observed when the compound was added post-transfection without any deleterious effect. Therefore, the addition of Pluronic F-68 acid post-transfection was maintained in future runs as these results suggested that, despite not having an impact on cell growth, the addition of Pluronic F-68 acid improved vector stability. In addition, in our batch process, the reduction of pH post-transfection from 7.2 to 6.8 showed higher cell productivities when FreeStyle 293 Expression medium was used during PEI:DNA complexation, similar to what was reported previously.^25^^,^^26^^,^^27^^,^^31^ However, other published work reported that pH as low as 6.0 destabilizes LVV particles^45^ and, thus, lower pH values were not explored in our work.

The highest LVV titers in the bioreactor were achieved when it was operated in perfusion, which was attributed to the viral harvesting at 4°C instead of 37°C. In a work undertaken by Higashikawa and Chang,^45^ the VSVG-pseudotyped LVVs were significantly less stable at 37°C or at room temperature vs. at 4°C. Their results demonstrated that at room temperature the half-life of the LVVs was between 1 and 2 days, rapidly reduced with increasing temperatures.

The VP/TU ratio is an indication of the quality of the LVV production. Ideally, a lower VP/TU is desired as this would imply a higher functionality of the LVVs produced. In our work, the VP/TU ratios were generally low compared with others studies published previously.^12^ It is possible that this is due to the short DSP performed to the virus harvested from the bioreactor in our work compared with others. In run 5, cell growth was lower compared with other bioreactor runs and the fact that the cell productivity was higher in this run was feasible as the amount of viral particles produced per cell may not be related to the total number of cells present during the transfection. However, a higher VP/TU ratio was observed in this particular run and this was the direct consequence of having lower cells in this instance. While it is not fully understood why lower cell densities have resulted in higher total VPs, it does demonstrate that cell growth is not the only factor that is important to consider for viral productivity.

In the cell and gene therapy field, it is important that the generated LVV particles are functional so they are capable of infecting and transducing the desired cell types. The focus, therefore, is to maximize functional LVV titers over total physical LVV particles. The final animal component-free LVV production process in the iCELLis Nano bioreactor in this work generated 1.2 × 10^6^ TU/cm^2^ with a total number of 6.4 × 10^9^ TU. These titers are higher than those reported previously in this same bioreactor using transient transfection with PEI and perfusion for the LVV production.^25^ In this study, 3.7 × 10^5^ TU/cm^2^ were achieved in a low-compaction 2.67 m^2^ fixed-bed bioreactor. In another study using also the 2.67 m^2^ fixed-bed of the iCELLis Nano bioreactor, a maximum of 6.7 × 10^5^ TU/cm^2^ were achieved.^26^ Finally, functional LVV titers of around 10^6^ TU/cm^2^ were reported in the iCELLis Nano bioreactor and the Scale-X platform by Leinonen et al.^10^ All previous studies reported were using DMEM supplemented with 10% FBS until at least 24 h post-transfection, compared with our work, which is fully animal component-free. Generally, functional LVV titers in literature are reported in TU/mL, making it hard to compare across different studies due to the innate variability in production scale in the different works. Only LVV production using adherent HEK293T cells should be reported in TU/cm^2^ as cells produce the virus attached to a surface. Furthermore, functional assays are not standardized and results are hard to compare across different laboratories.

In conclusion, these findings demonstrate that similar, if not better, functional LVV titers can be achieved when using FreeStyle 293 Expression medium compared with the conventional DMEM supplemented with 10% FBS process in both 2D culture and in the iCELLis Nano bioreactor. These results also demonstrate that the removal of animal-derived serum is not detrimental for the productivity of these cells in the bioreactor when FreeStyle 293 Expression medium is used. The developed chemically defined, animal component-free LVV production process may allow to reduce process cost, decrease batch-to-batch variability, and avoid the presence of animal-derived components in the final product. Moreover, the cost of viral vector manufacturing is greatly impacted by the final production titer; thus, an increased efficiency in the LVV production process will ultimately reduce the overall costs.

This work provides initial proof-of-concept demonstrating the feasibility of this platform using animal-derived serum-free medium to produce LVVs. Future studies could potentially optimize perfusion conditions to further increase production yields. In addition, it would be beneficial to explore run-to-run variability within the bioreactor and demonstrate the consistency of the platform. By demonstrating the feasibility and scalability of these processes, this study paves the way for more animal component-free production processes in the field of cell and gene therapy.

## Materials and methods

### Cell lines and cell culture

Adherent HEK293T cells (CRL-3216, ATCC, Manassas, VA) were cultured in high glucose DMEM (Gibco, Thermo Fisher Scientific, Waltham, MA) supplemented with 10% (v/v) FBS (Gibco, Thermo Fisher Scientific). Cells were thawed and cultivated in 12 mL of medium and seeded at 1 × 10^4^ cells/cm^2^ in T75 Nunc Cell Culture Treated EasYFlasks (Thermo Fisher Scientific).

Adherent HEK293T cells growing in DMEM supplemented with 10% FBS (serum-containing medium [SCM]) were adapted to FreeStyle 293 Expression medium (Gibco, Thermo Fisher Scientific) ( serum-free medium [SFM]) by sequential adaptation. In brief, cells were subcultured for at least two passages in each of the following medium compositions sequentially: 100% SCM, then 75% SCM + 25% SFM, then 50% SCM + 50% SFM, then 25% SCM + 75% SFM, then 10% SCM + 90% SFM, and then in 100% SFM for at least five passages before banking the cells.

Adherent, serum-free adapted HEK293T cells were cultured in FreeStyle 293 Expression medium without any additives or antibiotics. Cells were thawed and cultivated in 12 mL of medium and seeded at 1 × 10^4^ cells/cm^2^ in Corning CellBind T75 flasks (Sigma-Aldrich, St. Louis, MO). Cells were passaged when they reached 80%–85% confluence, usually every 3–4 days. In brief, medium was removed from the flask, cells were washed with 10 mL of phosphate-buffered saline (PBS) without calcium or magnesium (Lonza, Basel, Switzerland) and 5 mL of TrypLE Express Enzyme (Gibco, Thermo Fisher Scientific) were added on top of the cells. After 3–5 min of incubation at 37°C and 5% CO_2_, cells were harvested, pelleted, and re-seeded at the aforementioned concentration. Cells were cultivated at +37°C and 5% CO_2_.

### Lentiviral self-inactivating vectors

The transfer vector pALD-GFP and the three helper plasmids, pALD-Gag-Pol, pALD-VSVG, and pALD-REV, were purchased from Aldevron (Fargo, ND) at 1 mg/mL. The transfer vector encoding for a GFP had a spleen focus-forming virus promotor to drive the expression of the transgene cassette, followed by the GFP sequence, the presence of woodchuck hepatitis B virus post-transcriptional regulatory element encoding X protein, and finally an additional deletion in the 3′ LTR to “self-inactivate” the virus after the integration. The four plasmids were equipped with ampicillin resistance and were replication incompetent.

### Production of LVV particles in T75 flasks

HEK293T cells/cm^2^ (5 × 10^3^, 7 × 10^3^, or 2.5 × 10^4^) were seeded on day 0 in 12 mL of medium according to the seeding density of the experiment. For each different seeding density, three extra T75 flasks with the same number of HEK293T cells were seeded for cell counting on transfection day.

Third-generation self-inactivating (SIN) LVVs were produced by the transient transfection of four different plasmids into HEK293T cells with linear PEIpro (Polyplus, Illkirch, France). For the transfection, the DNA plasmid ratio transfer vector/Gag-Pol/REV/VSVG was 4:2:1:1.2, with the transfer vector encoding for a GFP protein.

Cells were then transfected 24, 48, 72, 96, 120, 144, or 168 h post-seeding according to the experiment. A total of 167 or 333 ng/cm^2^ of total plasmid DNA concentration, depending on the experiment, was used in combination with 1:2.75 DNA/PEI ratio (w/w). In brief, diluted PEI was added onto the diluted DNA and incubated for 15 min before being added on top of the cells. The DNA/PEI complexation volume corresponded to 5% of the final volume in the flasks and the medium used for DNA and PEI incubation was FreeStyle 293 Expression medium throughout all studies except in run 6, which was DMEM medium. Cells were then cultured for 48 h at +37°C and 5% CO_2_.

When the experiment required a medium exchange before transfection, the medium of each T75 flask of HEK293T cells to be transfected was replaced for fresh FreeStyle 293 Expression medium before addition of DNA/PEI complexes. When the medium exchange was performed post-transfection, the full flask medium was replaced for fresh medium 6 h post-transfection. If required in the experiment condition, 5 mM sodium butyrate (Sigma-Aldrich), 1× non-essential amino acids (Gibco, Thermo Fisher Scientific), 1× chemically defined lipids (Gibco, Thermo Fisher Scientific), or 1 mM sodium pyruvate (Gibco, Thermo Fisher Scientific) was added to the culture medium.

Forty-eight hours post-transfection, the medium containing the LVVs was collected, centrifuged for 5 min at 300 × *g* at 4°C to remove any cell debris, and the supernatant was then filtered through a 0.45 μM polyethersulfone (PES) filter. Aliquots were frozen and stored at −80°C.

### Operation in the iCELLis Nano bioreactor

The iCELLis Nano bioreactor (Pall Corporation, Port Washington, NY) is a bench scale, fixed-bed bioreactor ideal as a process development tool. It consists of a cylindrical bed with randomly packed proprietary macrocarriers made of microfibers of polyethylene terephthalate. The fixed-bed is available in six different surface areas ranging from 0.53 to 4 m^2^, either in low (96 g/L) or high compaction (144 g/L) carrier configuration. Each macrocarrier has a culture area of 13.9 cm^2^.

Low-compaction, 0.53 m^2^ fixed-beds were used for the work described below. The autoclaved vessel was filled and equilibrated overnight with 700 mL of DMEM supplemented with 10% FBS (v/v) or FreeStyle 293 Expression medium depending on the experiment. During the equilibration, stirring was set at 2 cm/s medium linear speed. A cell suspension of 150 mL with 1.3 × 10^8^ or 2.7 × 10^7^ total HEK293T cells (2.5 × 10^4^ or 5 × 10^3^ cells/cm^2^, respectively) was inoculated the next morning to the bioreactor. The stirring was maintained at 2 cm/s for 5 h post-inoculation, before being decreased to 1 cm/s for the rest of the experiment. The final working volume was 850 mL, equivalent to the volume per cm^2^ used in the control flasks.

The temperature set point was 37°C. The dissolved oxygen set point was 50% air saturation, maintained with stirring and headspace air or oxygen supply. The pH set point was 7.2 during the HEK293T cell expansion phase controlled by headspace CO_2_ addition. For runs 1 to 4 the pH was also controlled with 7.5% sodium bicarbonate (Gibco, Thermo Fisher Scientific). In runs 5 to 8, the pH post-transfection was decreased to 6.8. Daily samples were taken for offline measurements of cell counts in the supernatant, pH, glucose, lactate, and ammonia. Online pH was monitored using daily offline measurements with the SevenCompact pH meter S220 (Mettler Toledo, Greifensee, Switzerland). Metabolite measurements were determined with the Optocell CuBiAn HT-270 (4BioCell, Bielefeld, Germany). Runs 1 to 6 were run in batch mode and runs 7 and 8 were run in perfusion at 1 vvd.

### Production of LVV particles in the iCELLis Nano bioreactor

Third-generation SIN LVVs were produced by the transient transfection of four different plasmids into HEK293T cells using PEI 48 h (day 2) or 120 h (day 5) post-inoculation, depending on the experiment. The optimized LVV production conditions at small scale were used in the LVV production in the bioreactor. In brief, a total of 167 or 333 ng/cm^2^ of total plasmid DNA concentration were used in the experiments in combination with 1:2.75 DNA/PEI ratio (w/w). The DNA/PEI complexation volume corresponded to 5% of the working volume in the bioreactor and was incubated for 15 min before being added to the cell suspension. The DNA plasmid ratio transfer vector/Gag-Pol/REV/VSVG was 4:2:1:1.2, with the transfer vector encoding for a GFP gene.

When the experiment required a medium exchange before transfection, the whole bioreactor volume was replaced with fresh FreeStyle 293 Expression medium before addition of DNA/PEI complexes. When the medium exchange was performed post-transfection, the whole bioreactor volume was replaced 6 h post-transfection with 850 mL of medium supplemented with 5 mM of sodium butyrate and 0.1% Pluronic F-68 acid (Gibco, Thermo Fisher Scientific) if required by the experiment conditions.

In the experiments run in batch mode, the medium containing the LVVs was collected 48 h post-transfection, centrifuged for 5 min at 300 × *g* at 4°C to remove any cell debris, and the supernatant was then filtered through a 0.45 μM PES filter. Viral supernatant aliquots were frozen and stored at −80°C.

In the runs where perfusion was used, this was started 6 h post-transfection and the outlet perfusion bottle was kept in a bucket with ice to maintain the LVV at 4°C. The inlet perfusion bottle with fresh medium was kept at room temperature. LVV harvest started 6 h post-transfection after the medium exchange until 48 h after transfection. Perfusion was performed with medium supplemented according to the experiment at 1 vvd.

A control flask was run in parallel for each of the runs using the same culture and transfection conditions; including the same inoculation density, the same total plasmid DNA concentration (ng/cm^2^), and same medium volume per surface (0.16 mL/cm^2^).

### Nuclei cell count in the iCELLis Nano bioreactor

Cell growth in the bioreactor was monitored daily for the whole duration of the runs. Three macrocarriers from the top of the fixed-bed were sampled every day through the biomass probe port, resuspended in 1.5 mL of Reagent A100 (Chemometec, Allerod, Denmark), and vortexed for 2 min for cell lysis. Macrocarriers were then discarded and nuclei from the lysed cells were counted using the NucleoCounter NC-200 (Chemometec). Three macrocarriers from the top, middle, and bottom of the fixed-bed were also counted at the end of the culture after the last harvest to assess potential growth differences across the fixed-bed.

### Determination of functional and total physical LVV titers

HEK293T cells (1 × 10^5^) supplemented with 10% (v/v) FBS were seeded in 1 mL per well in a 12-well plate (Thermo Fisher Scientific) the day before transduction. Three extra wells with the same number of cells were seeded for cell count on day of transduction, 24 h after seeding. To estimate viral titers, serial dilutions were prepared with each unconcentrated viral supernatant and high glucose DMEM to a final volume of 1 mL. To each aliquot, 8 μg/mL of Polybrene (Santa Cruz Biotechnology, Dallas, TX) was added to enhance transduction. The volume of each well of the 12-well plate was replaced with 1 mL of the aliquot and the cells were spinoculated at 1,000 × *g* for 1 h 30 min at 32°C with the break off. Medium was replaced after 20–24 h for 1 mL fresh high glucose DMEM and cells were incubated for 48 h.

Seventy-two hours after transduction, medium from each well was removed and cells were harvested with TrypLE Express Enzyme following the manufacturer’s recommendations. Cells were then centrifuged at 300 × *g* for 5 min, resuspended to a final volume of 500 μL with PBS and 5 μL of 7-AAD (BioLegend, San Diego, CA) were added to the cells. After 10 min of incubation at room temperature in dark, viable GFP^+^ cells were analyzed by flow cytometry (BD Biosciences, Franklin Lakes, NJ). Functional LVV titers were calculated using the following formula:

$$\frac{\left(Total\;number\;of\;cells\;at\;the\;moment\;of\;transduction\right)\times \left(\frac{\%\;live\;{GFP}^{+}\;cells}{100}\right)}{Virus\;volume\;\left(ml\right)}$$

For the evaluation of the total physical LVV particles generated in the bioreactor, p24 capsid protein was measured using the QuickTitre Lentivirus Titer Kit from Cell Biolabs (San Diego, CA) in duplicates following the manufacturer’s recommendations. It was assumed that in 1 ng of p24 there are 1.25 × 10^7^ lentiviral particles based on the kit manufacturer’s recommendations.

### Statistical analysis

The statistical analysis was performed using FlowJo 10 (BD Biosciences) and GraphPad Prism 9.4.0 (GraphPad Software, San Diego, CA) software. Statistical analysis was performed using ordinary one- or two-way ANOVA with Tukey’s multiple-comparisons test. Statistical significance was determined when the p value was ≤0.05 compared with the control condition or to another condition stated in the figures.

## Data availability

All data generated or analyzed during this study are included in the article.
